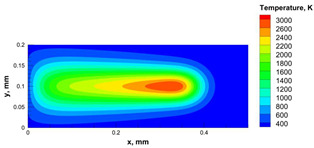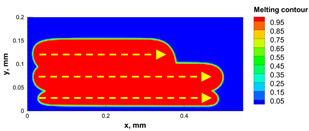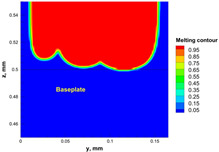# Multi-Layer Simulation of the Powder Bed Selective Laser Processing of Alumina for Residual Stress and Distortion Evaluation

**DOI:** 10.3390/ma15103498

**Published:** 2022-05-13

**Authors:** Mohamed Abdelmoula, Gökhan Küçüktürk

**Affiliations:** Department of Mechanical Engineering, Gazi University, Ankara 06500, Turkey; mohamedeid@gazi.edu.tr

**Keywords:** selective laser processing, alumina, CO_2_ laser, build orientations

## Abstract

A numerical model was developed to simulate the real process of alumina powder bed selective laser processing (PBSLP) to thoroughly investigate the residual stress and distortion experienced in printed parts when multi-layer scanning with a CO_2_ laser source is considered. The model contains a user-defined function (UDF) for the laser source, temperature-dependent material properties, scanning strategies, and build orientations, and it is solved using ANSYS 2020R2. In addition, the model’s validation was confirmed with experimental results. The results revealed that a high scanning speed (up to 1200 mm/s) and low laser power are effective for the PBSLP of alumina, owing to alumina’s high absorptivity for CO_2_ lasers, and a high manufacturing rate can be achieved. During the multi-layer printing simulation, the accumulated heat inside the part increased gradually with an increased number of printed layers. Additionally, the calculated residual stress exceeded the yield limit for all the studied build orientations due to the printed part’s high-temperature difference. When preheating was applied, the residual stress decreased by 23% and the distortion decreased by 54%. For the successful PBSLP of ceramics, commercial printers cannot be used effectively. A particular printer equipped with a temperature controller and a preheating system is required for ceramics.

## 1. Introduction

Ceramic materials represent one of the most important material classes, with numerous applications in a variety of fields due to their distinct mechanical and physical properties [1]. These properties include low density, high hardness, biocompatibility, and corrosion resistance [2,3,4,5]. Ceramic parts are manufactured using traditional methods such as casting, extrusion, injection molding, and pressing [6,7,8,9]. The problem with these techniques is that they cannot keep up with the current manufacturing revolution that involves the production of highly complex designs. Furthermore, ceramic parts produced using these traditional techniques require post-treatment operations to achieve the final shape, which incurs additional costs and creates problems such as shrinkage [10,11].

Additive manufacturing (AM) technology has the potential to be an effective solution because of its ability to produce highly complex designs [12,13,14]. AM is a trending manufacturing technology which is used to produce parts from 3D CAD models layer by layer [15]. AM has seven techniques defined by ISO/ASTM 52900, i.e., powder bed selective laser processing (PBSLP), binder jetting, vat-photopolymerization, extrusion, direct energy deposition, material jetting, and sheet lamination [15].

Nowadays, the AM of ceramics is undergoing rapid developments, whether in terms of feedstock or the application of AM techniques. Many studies have been conducted on the AM of ceramic materials using various techniques such as binder jetting, extrusion, and PBSLP [16,17,18,19,20,21,22,23,24,25,26,27,28,29]. Among the techniques used, PBSLP is regarded as the most suitable for ceramic materials because it can produce a dense structure with more accurate shape dimensions and without the need for initial powder or post-treatment operations to achieve the final shape [30]. However, many problems hinder the optimal application of this technique for the AM of ceramics, as in the case of metal materials. These problems are a high melting/sintering point, low thermal conductivity, and laser absorptivity [31,32,33,34].

The high melting/sintering point of ceramics causes high thermal shock during the PBSLP process, when the temperature increases from room temperature to more than 2000 K in a very short time [35,36]. The low thermal conductivity of ceramics hinders the diffusion of the heat generated from the laser melting/sintering process through the powder layer. This leads to nonhomogeneous heating of the layer, and as a result, cracks generate because of the developed thermal stresses, which exceed the yield limit of the material [30,37].

Ceramic materials are well-known for absorbing light energy with varying wave-lengths depending on their optical properties [38,39]. For example, the absorptivity of oxide ceramic is very high with a 10.64 µm wavelength and very low with a 1.064 µm wavelength, but carbide ceramic behaves in an opposite manner. Thus, oxide ceramic’s best light energy source is a high wavelength such as a CO_2_ laser. A study by Pham et al. [39] supported this idea, in which alumina and silicon nitride milling were studied using an Nd-YAG laser with a 1.064 µm wavelength. They found that the machining accuracy was highly dependent on laser absorptivity.

Several previous studies have focused on the PBSLP of ceramics to overcome the described difficulties. Liu et al. [33] investigated the effect of high-temperature preheating on the PBSLP of yttria-stabilized zirconia (YSZ) ceramic to overcome the thermal stresses and cracks that develop during the PBSLP. They found that preheating could effectively reduce the developed cracks. Hagedorn et al. [31] used a CO_2_ laser preheating system to heat up the deposited layer before scanning with a Nd-YAG laser to control the cracks. They found that cracks were reduced, particularly as the preheating temperature was increased. Zheng et al. [35] experimentally investigated the effects of scanning strategies on developed cracks during the PBSLP of alumina using island and zigzag scanning strategies. They found that the zigzag strategy resulted in both transverse and longitudinal cracks, whereas the island strategy only resulted in transverse cracks. Additionally, Liu et al. [40] investigated the effects of laser powers and scanning speeds on the microstructure and density of YSZ parts produced with PBSLP. They found that when the laser energy density was insufficient to melt/sinter the powder particles, many pores formed inside the sample, influencing mechanical performance and density. It has, however, become a plentiful source of crack initiations.

Other studies have focused on the difficulty related to laser absorptivity in ceramic materials. Enrique et al. [36] mixed alumina with graphite and used a commercial SLM printer for the PBSLP of alumina powder. The powder absorptivity was increased to more than 50%. Segado et al. [41] investigated adding graphite to hydroxyapatite and chlorapatite powders to increase laser absorptivity and found that adding graphite to the mixture enlarged its processing window.

According to the presented literature review, researchers have carried out numerous experimental trials and have spent considerable time determining the best process window for the PBSLP of studied ceramic materials. Furthermore, previous numerical studies on the PBSLP of ceramic materials have investigated either a single-track [27,28,29,42,43,44,45] or one-layer simulation [46]. Using numerical modeling to predict the process output is a powerful tool and should be considered in AM to obtain a general view about the effect of the process parameters [47,48,49]. Concerning the laser heat source, several previous studies [29,35,36,42] have focused on using either fiber or Nd-YAG lasers for the PBSLP of alumina, despite the fact that the CO_2_ laser is considered to be the best choice based on its interaction with alumina.

The aim of this study was to develop a numerical tool that could simulate the real multi-layer PBSLP and to conduct an in-depth investigation of the melting/sintering process of ceramic materials using a CO_2_ laser as the heat source. Furthermore, the developed model should take into consideration the thermal stress and distortion calculations for alumina PBSLP.

## 2. Materials and Methods

### 2.1. Numerical Procedure

It is worth mentioning that the process of numerically modeling the melting/sintering of the powder particles is complex. When the laser system starts scanning the powder layer, part of the laser energy is used to process the powder particles, while the other part is reflected into the printer chamber. Therefore, several assumptions were considered: (1) the powder bed is homogenous and continuous media, (2) the molten pool top surface is flat, (3) the laser heat source is uniformly distributed, and (4) no heat losses are considered to be due to evaporation. In addition, heat transfer applied by conduction, radiation, and convection was considered during the development of the model.

#### 2.1.1. Numerical Model Development

A sintering/melting model was developed to simulate the multi-layer PBSLP process as follows: Initially, the layer to be printed is deposited, and is modeled as alumina powder; all the layers above this layer are modeled as an inert gas. Afterwards, the process parameters (scanning speed, scanning strategy, laser power, and hatching distance) are applied, and the scanning of this current layer continues until it is completed. The transition from the current completed layer to the next layer waits until the spreading of a new layer. Next, the material properties of the gas layer, which is above the previously scanned layer, are changed from inert gas properties to alumina powder properties. Finally, all the above steps are automatically repeated through the developed model until the entire part has been simulated.

The developed numerical model contains a UDF for the scanning strategies, build orientations, a laser heat source, and material properties, according to Moser et al. [50]. In addition, solidification and melting calculations are also included in the model to monitor the melted phase of the material.

The heat transferred to the powder from the laser source can be modelled by the energy equation as follows [50,51]:

(1) $$\rho C_{p}\frac{\partial T}{\partial t}=\nabla \cdot \left(K\;\nabla \;T\right)+S_{h}$$

where $\rho \;,C_{p}\;,T$, and K are the density, specific heat, temperature, and thermal conductivity, respectively. For alumina powder, the thermal conductivity, specific heat, and other properties are summarized in Table 1 as a function of temperature.

**Figure 1:**
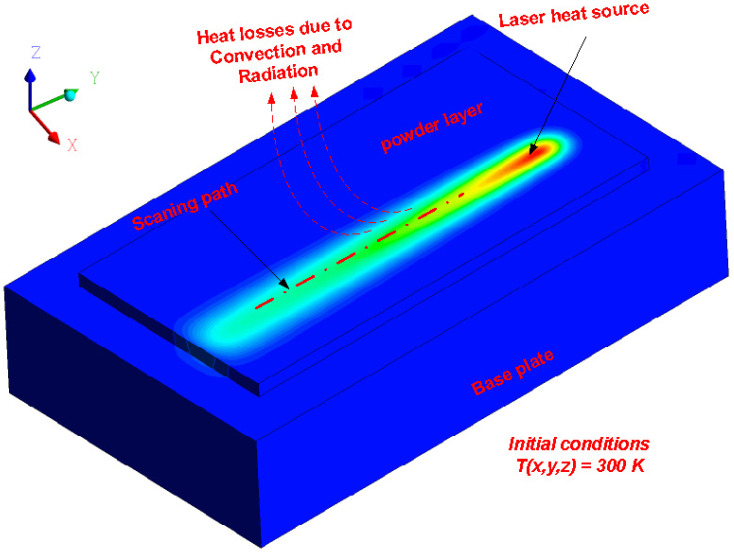
Initial and boundary conditions considered during the model development.

The heat source representing the laser beam is considered in Equation (1) by the heat source term $S_{h}$ and has a Gaussian distribution profile according to [50]. It can be described as follows:

(2) $$S_{h}=AI_{o}\alpha \;\exp\left(-2\;\frac{{\left(x-v_{x}t\right)}^{2}+{\left(y-v_{y}t\right)}^{2}}{\omega^{2}}-\alpha z\right)$$

where *A* represents the material laser absorptivity; α represents the effective absorption coefficient; $I_{o}$ represents the laser heat intensity; ω represents the characteristic radius of the laser spot; and *x*, *y*, and *z* represent the location of the laser spot size of the powder layer. According to [50]:

(3) $$I_{o}=\frac{2P}{\pi \;\omega^{2}}$$

(4) $$\omega =\frac{D_{b}}{2\times 2.146}$$

where P is the laser power and $D_{b}$ is the laser spot size. The initial condition and the boundary conditions used in this study are according to Equations (5) and (6), respectively [45], and Figure 1 shows the boundary conditions applied in the developed numerical model.

(5) $$T{\left(x,y,z\right)}_{t=0}=T_{O}$$

(6) $$-k(\frac{\partial T}{\partial z})=\dot{S_{h}}-h_{cov}\left(T_{a}-T_{s}\right)-\sigma \varepsilon \left(T_{a}^{4}-T_{s}^{4}\right)$$

where $T_{O}$ is the chamber initial temperature (300 K),$\;h_{cov}$ represents the convection coefficient, $T_{a}$ is the initial temperature of the powder layer, $T_{s}$ is the surrounding medium temperature, ε represents the emissivity, and σ represents the Stefan–Boltzmann constant. All other material constants are summarized in Table 1.

The enthalpy technique described by [51] was used to model the sintering and solidification during the PBSLP process. This technique depends mainly on the material enthalpy, defined as the total heat content in the system, the sum of the internal energy, and the pressure and volume product, as described by Equation (7). In addition, the enthalpy equals the sensible heat and latent heat content in the system, as defined by Equation (8).

(7) H=U+PV

(8) H=h+ΔH

where *U* represents the internal energy of the system, *P* represents the system pressure, *V* represents the volume change, *h* represents the sensible heat, and Δ*H* represents the latent heat. According to [51], *h* and Δ*H* can be expressed as follows:

(9) $$h=h_{ref}+C_{p}\;\Delta T$$

(10) ΔH=βL

where *h_ref_*, L, and β represent the reference enthalpy, the latent heat, and the liquid fraction, respectively. The liquid fraction β can be estimated as follows [51]:

(11) $$\beta =\frac{T-T_{solidus}}{T_{\mathrm{liquidus}}-T_{solidus}}.$$

The temperature *T* can be calculated by solving Equation (1), and then used to measure β, which defines the melting or solidification within the solution domain according to Equation (12):

(12) $$\beta =\{\begin{array}{c} <1\\ =0\\ >1 \end{array}\begin{array}{c} solid\;region\\ transition\;region\\ melting\;region \end{array}$$

A finite element analysis (FEA) was used to calculate the residual stress and distortion developed in the printed part though a coupled thermal-mechanical analysis. The FEA is mainly based on the relation between the developed stress and strain. Firstly, this relation is considered to be a linear relation (elastic region), and after yielding, it becomes a nonlinear relation (plastic region). It is worth mentioning that the bilinear plasticity model was used to describe the relationship between stress and strain as described in [52].

The mechanical properties of alumina used for the residual stress and distortion calculation are expressed as a function of temperature as follows [53,54,55,56]:

(13) $$E=407.1-7.3407\times 10^{-2}T$$

(14) $$\alpha =-0.23036+7.0045\times 10^{-4}\;T+5.681\times 10^{-8}T^{2}$$

(15) $$\sigma_{y}=-0.154\;T+306$$

where *E* is the elastic modulus (GPa), α is the thermal expansion coefficient (10^−6^·K^−1^), $\sigma_{y}$ is the yield stress (MPa), and T is the temperature in K.

#### 2.1.2. Numerical Model Geometry

**Figure 2:**
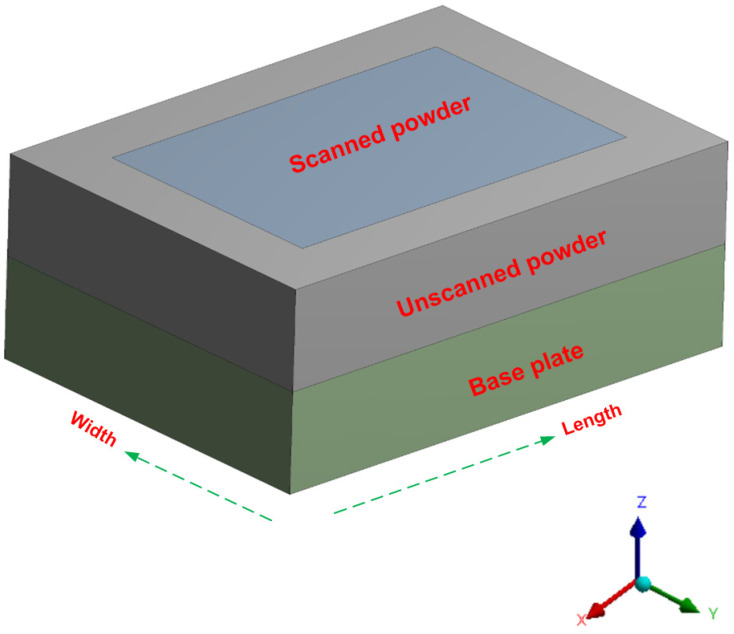
The model geometry used in the analysis (not to scale).

Figure 2 shows the numerical model geometry used in this analysis, and the dimensions are summarized in Table 2. The numerical model geometry consists of the base plate, the laser working space, and the unscanned surrounding powder. The model was generated using the ANSYS Design Modeler.

**Figure 3:**
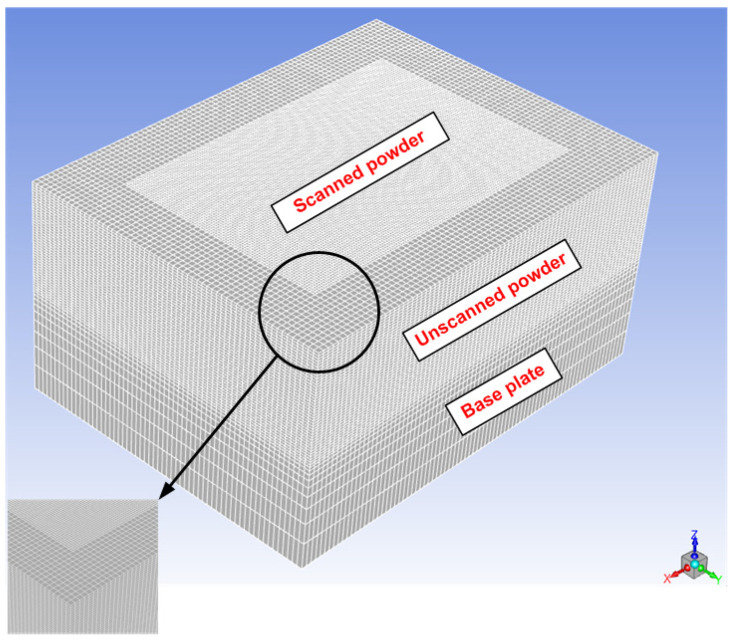
The mesh (computational domain) used in this study (not to scale).

Figure 3 shows the computational domain used in this study where ANSYS Mesher was used to create the computational domain (the mesh). Very fine discretization was considered during the computational domain generation, especially for the scanned powder region.

**Figure 4:**
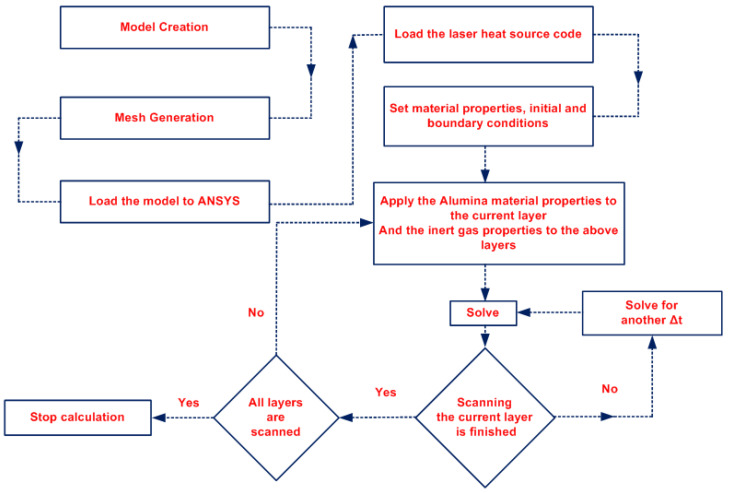
The numerical model solution steps.

In order to avoid any errors coming from bad quality meshing, a grid independence test was conducted to evaluate four different mesh sizes, i.e., 10, 5, 2.5, and 2 µm. The maximum temperature was used to evaluate the effect of mesh density, and the results are summarized in Table 3. The maximum temperature became stable after the mesh size of 5 µm. Therefore, the 5 µm mesh size was used to reduce the calculation time. Figure 4 presents the steps followed during the numerical model solution.

#### 2.1.3. Alumina PBSLP Using the CO_2_ Laser

The PBSLP of alumina was investigated, considering a CO_2_ laser as the melting source. Different power values and scanning speeds were investigated to determine the range of the laser power and scanning speed that could be used successfully for the PBSLP of alumina. Table 4 summarizes the range of the laser power, scanning speed, and other process parameters investigated in this study.

**Figure 5:**
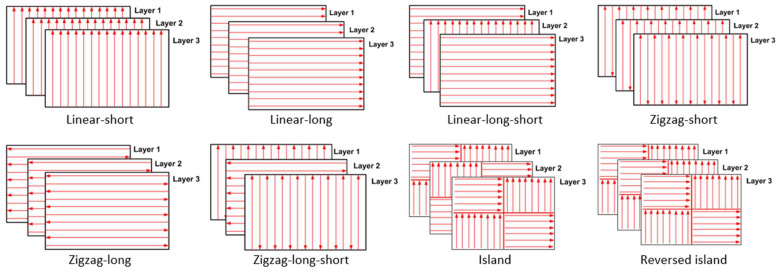
Build orientations used in this study.

Additionally, build orientation is another crucial parameter for the PBSLP of alumina as it directly affects the mechanical properties of the printed part. Figure 5 shows the build orientations which were investigated in this study.

### 2.2. Experimental Procedure

**Figure 6:**
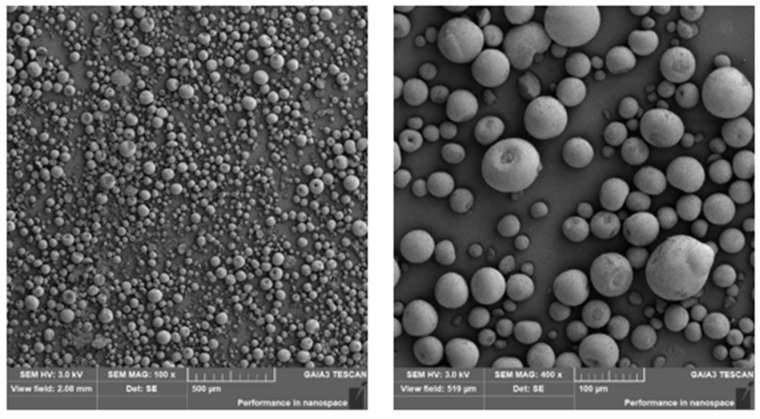
SEM images of spray dried alumina powder [29] (republished based on the open access permission).

The commercial SLM 125 printer, manufactured by RENISHAW^®^ (Wotton-under-Edge, England), was used to print the alumina parts and validate the numerical model. This printer is equipped with a fiber laser source, a wavelength of 1070 nm, a maximum laser power of 200 W, and a spot size of 70 µm. The process parameters used to produce the alumina samples, i.e., a cube shape with a side length of 10 mm, were as follows: a layer thickness of 100 μm, a laser spot size of 70 μm, a hatching distance of 50 μm, and the zigzag scanning strategy. Alpha-alumina powder with a purity of 99.7%, supplied by Alteo, was used as the feedstock. Due to the low absorptivity of alumina to fiber lasers, the alumina powder was mixed with 0.1 vol% colloidal graphite to increase the absorptivity. A previous study described the mixing process in detail [36]. In Figure 6, the SEM images of spray-dried alumina powder with a d_50_ size of 39.8 µm are presented.

## 3. Results and Discussion

### 3.1. Numerical Model Validation

A numerical model was used to calculate and determine the laser power and scanning speed that could achieve full melting of the alumina layer thickness and give a melting temperature below the evaporation point of alumina. The alumina samples were printed and used for the model validation. Based on our previous study [29], it was recommended to use a low scanning speed with alumina to minimize the laser beam inertia effect on the powder particles. Therefore, a scanning speed of 200 mm/s was considered. Table 5 summarizes the results obtained from the numerical model. It was found that using a laser power ranging from 95 to 105 W satisfied the abovementioned conditions.

**Figure 7:**
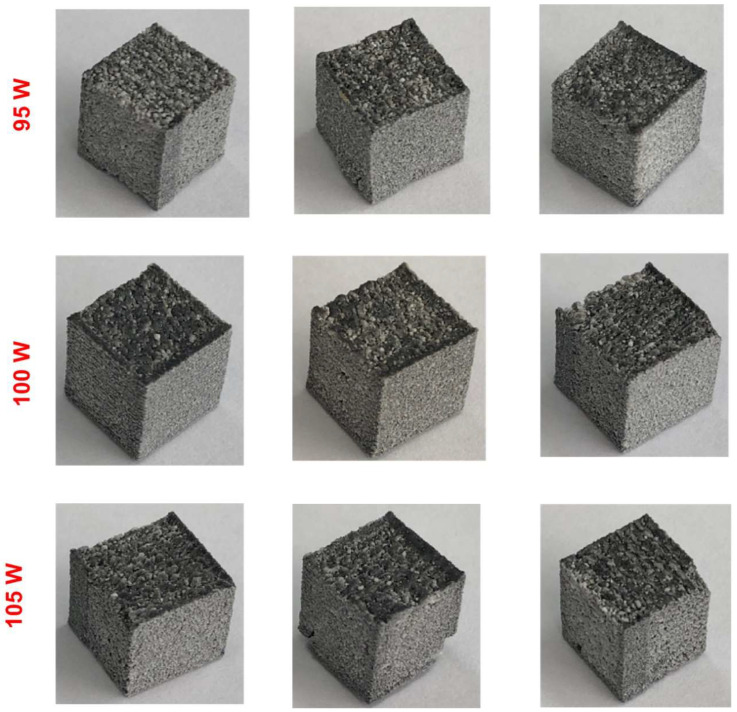
Alumina cubes printed by using the process parameters calculated from the model.

Additionally, the width of the melting path ranged from 65 µm to 75 µm for the calculated laser powers. Therefore, a hatching distance of 50 µm was used to connect the adjacent paths. Different alumina samples were printed successfully using the process parameters obtained from the numerical model (Figure 7). Additionally, these successfully printed samples confirmed the validation of the developed numerical model and proved the developed model’s ability to correctly estimate the appropriate values of the process parameters.

**Figure 8:**
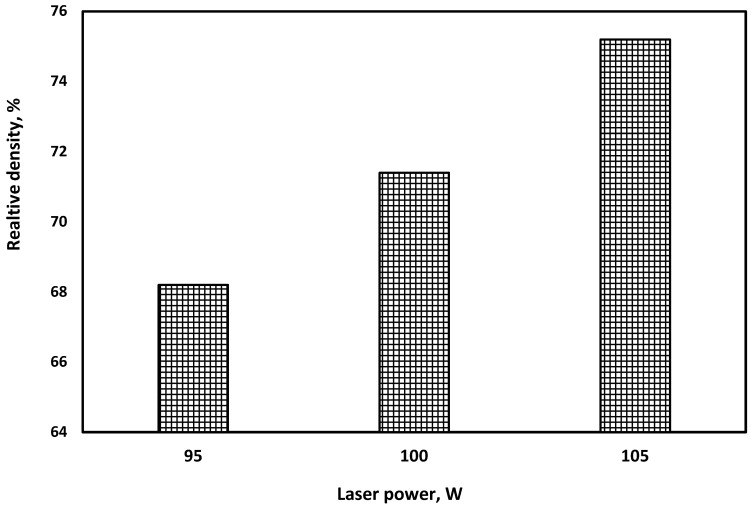
Alumina samples’ relative density results.

Measurements based on Archimedes’ principle were made to evaluate the relative density of the printed samples, and the results are provided in Figure 8. The prominent results were examined; a relative density of 68% was obtained when a 95 W laser power was used, while it was possible to measure up to 75% of the relative density when the laser power was increased to 105 W. The resulting relative density can be considered a low relative density. The main reason for this is thought to be the relatively thick layer thickness. The layer thickness used was 100 µm, and we could not decrease it due to the used printer’s equipment capabilities. Using a low layer thickness and a compaction system for the powder layer are suggestions to increase the relative density.

**Figure 9:**
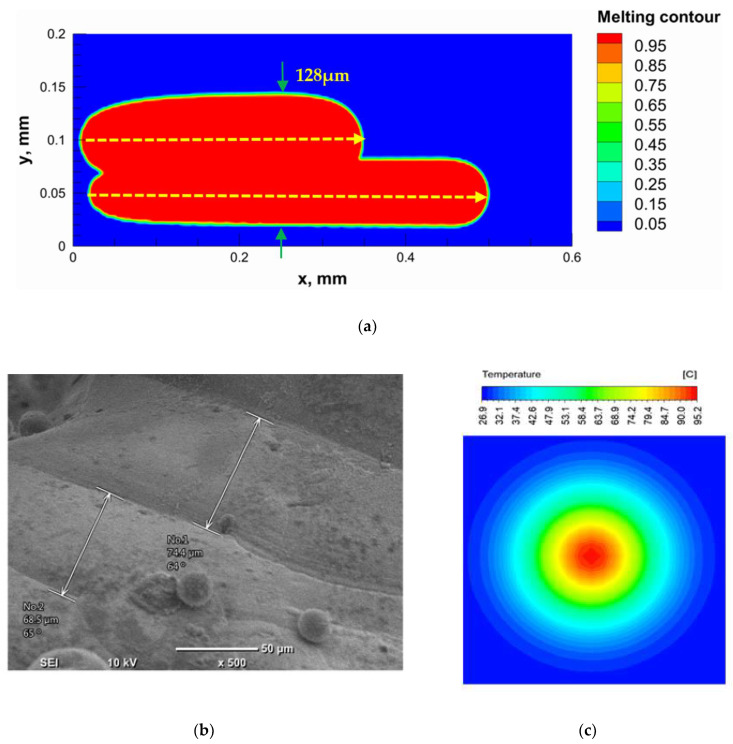
Validation of the experimental data with numerical model: (**a**) SEM images for two adjacent melting paths; (**b**) melting contour for two adjacent melting paths; (**c**) temperature distribution for the laser spot obtained from the numerical model.

The numerical model was validated, at the micro-scale level, by comparing the melting path width experimentally and numerically. SEM images were used to measure the melt path width experimentally, which was 142.9 µm, as shown in Figure 9a; a melt path width of 128 µm for the two adjacent paths was obtained from the numerical model (Figure 9b). It can be concluded that the two measurements were very close, with a calculation error of 8%, which confirms the validation of the developed numerical model.

Since the developed numerical model was used for the residual stress calculation, it was crucial to ensure that the obtained temperature distribution from the model was correct and validated. Therefore, the temperature distribution obtained from the developed numerical model was compared with the available experimental data [50,57]. The laser spot temperature contour data were captured using a thermal camera (TVS-2300ST, Avio Nippon Avionics Co., Ltd., Kanagawa, Japan). It was found that the laser spot temperature contour captured by the thermal camera [50,57] and the laser spot temperature contour obtained from the numerical model at the same conditions (Figure 9c) showed good agreement with a maximum error of 1.24% between the two contour temperatures.

### 3.2. Process Window for the PBSLP of Alumina Using the CO_2_ Laser

**Figure 10:**
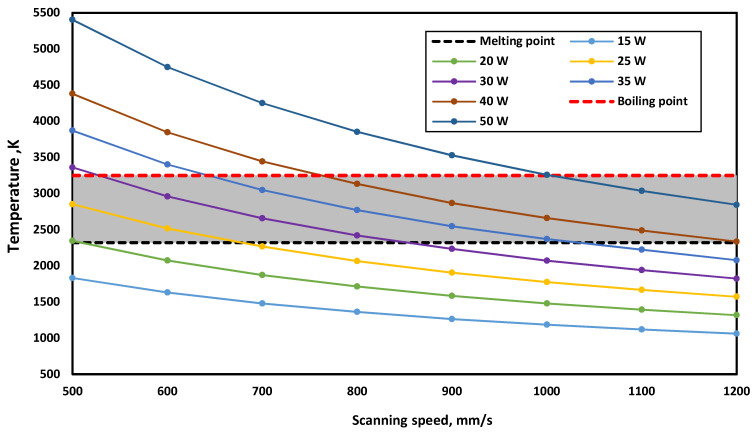
Obtained maximum temperature at different powers and scanning speeds using the CO_2_ laser.

The developed model was used to determine the appropriate laser power values at each scanning speed based on the obtained temperature that should lie between the melting and boiling point of alumina. Figure 10 shows the temperature values at different powers with different scanning speeds using the CO_2_ laser. All the laser power values with scanning speeds that give a temperature value in the grey region can be used for the PBSLP of alumina. The values below this region cannot achieve melting, and the values above it can cause boiling of the material and evaporation. It can be observed that the laser power values are low, and the scanning speed values used with the CO_2_ laser are very high compared with the values used with fiber or Nd-YAG lasers as described in previous studies [36,42]. This can be attributed to the absorptivity of alumina for both the Nd-YAG laser and the CO_2_ laser, where the absorptivity of alumina to the Nd-YAG laser is very low, reaching 3%, while for the CO_2_ laser, the absorptivity is very high, reaching 96%. Therefore, using a CO_2_ laser can save laser power and increase the manufacturing rate of the PBSLP of alumina. Additionally, no powder preparation is needed to increase the powder absorptivity using a CO_2_ laser.

To confirm that the values of the laser power and scanning speed, which give temperature value within the grey region, could melt the layer thickness and produce connected scanning paths, the developed model was used to investigate different values, as can be seen in Table 6. The tested values gave connected scanning paths and could melt the whole layer thickness.

### 3.3. Temperature History during Part Printing

The temperature history during scanning is critical to obtain a comprehensive view of the laser scanning process. The process window shown in Figure 10 was used to select the appropriate laser power and scanning speed in order to investigate the temperature history generated during the PBSLP of alumina. A laser power of 50 W, a scanning speed of 1200 mm/s, and a hatching distance of 50 µm were selected. The numerical model developed was solved using these process parameters and the build orientations depicted in Figure 5.

**Figure 11:**
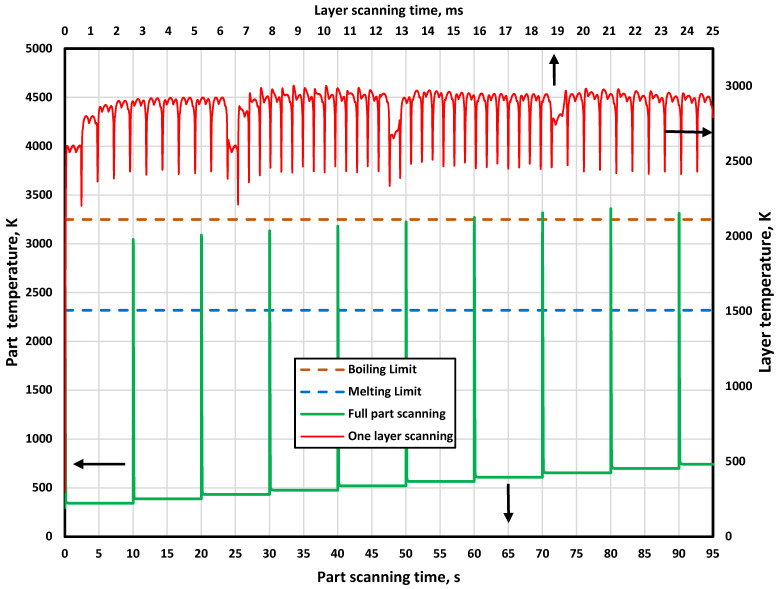
The temperature history for the full part and one layer printing using island scanning strategy.

Figure 11 shows the temperature history during the printing process for the whole-part scanning using the island scanning build orientation (the part contains 10 layers). It also shows the temperature history for one-layer scanning obtained from the developed multi-layer PBSLP model. The green curve shows the temperature history for the whole-part scanning while the red curve shows the temperature history for the one-layer scanning. The temperature and scanning time axes for the whole-part scanning are positioned at the right and the bottom of the figure, respectively, whereas, for the one-layer scanning, they are positioned at the left and top of the figure, respectively.

The laser beam started to scan the first layer at a scanning time of 0 s, and it took 0.025 s to finish the scanning. The scanning time was very short as the dimensions of the layer were small, as described in Table 2. During the first layer scanning, the temperature history was between the melting and boiling limits. After finishing the first layer scanning, the temperature went down to above room temperature After finishing the first layer scanning, the temperature went down to above room temperature, which means there was heat accumulation inside the part, and the laser beam waited for the deposition of the second layer. The deposition time was set to be 10 s, which can be adjusted according to the specifications of the used printer, shown in Figure 11. After the second layer deposition, the laser beam started scanning. All the previous steps were repeated until finishing all layers.

By following the temperature history for the whole-part scanning, it is obvious that the temperature history increased gradually with the deposition and the scanning of consecutive layers. This was mainly due to the gradually increasing heat accumulated inside the printed part. The heat accumulation increased gradually inside the part due to the low thermal conductivity of alumina and the little time available for releasing the heat away from the part. The accumulated heat inside the part caused the temperature of the last four layers to exceed the boiling point (as shown in Figure 11). This could lead to defects in the form of porosities and cracks. Additionally, unlike the observed trend in all layers, the temperature history of the last layer was less than the previous layers, and this was mainly because the last layer had sufficient time to release the heat as no powder was deposited above it to keep the heat inside.

It can be concluded from monitoring the temperature history that PBSLP printers for ceramic materials should be equipped with a temperature controller to hold the temperature between the melting and boiling limit during the printing process. The temperature can be adjusted by controlling either the laser power or the scanning speed. The temperature history for the other build orientations is almost the same as the island scanning build orientation and, therefore, was not presented.

### 3.4. Residual Stress and Distortion

**Figure 12:**
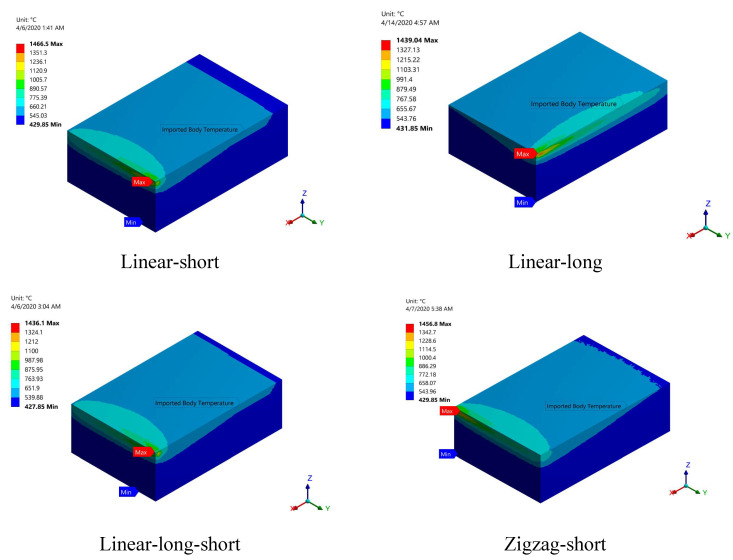

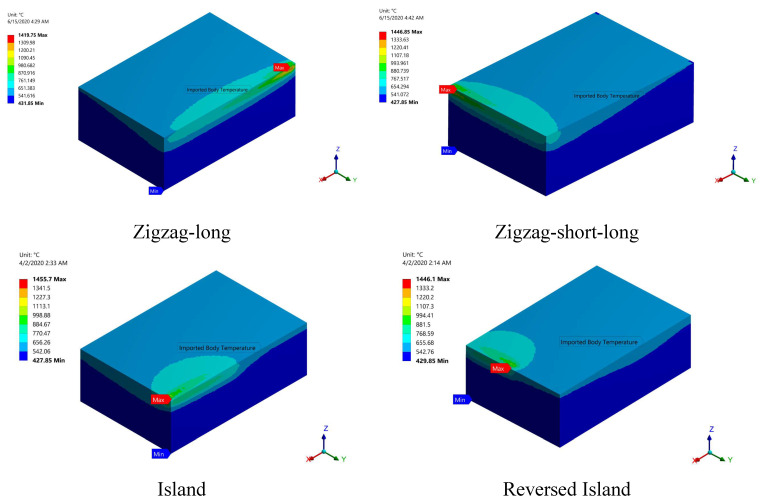
Temperature contours for the studied build ordinations just after solidification.

The residual stress calculation is entirely dependent on the temperature distribution of the printed part. To calculate the residual stress for each build orientation, the temperature distribution was used as a thermal load in the coupled thermal-mechanical FEA model. Figure 12 depicts the temperature distribution obtained from the multilayer PBSLP model for each build orientation just after the printed part solidifies. The temperature distribution reflects the scanning strategy used in each build orientation, as indicated by the last scanned layer.

The von Mises equivalent stress criterion (σ_e_) was used to investigate and assess the residual stress developed in the printed parts. Because the thermal and mechanical properties of the printed part, particularly the yield stress (σ_y_), vary with temperature, the von Mises equivalent should be normalized by dividing the von Misses equivalent stress at a specific point by the yield stress at the same point.

**Figure 13:**
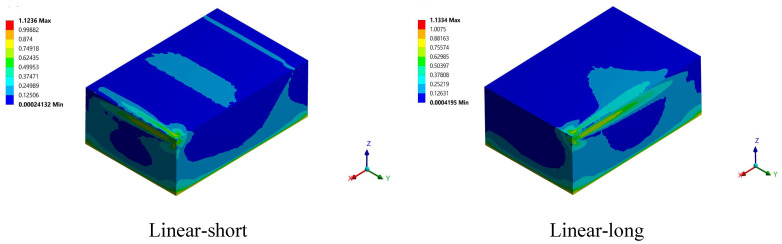

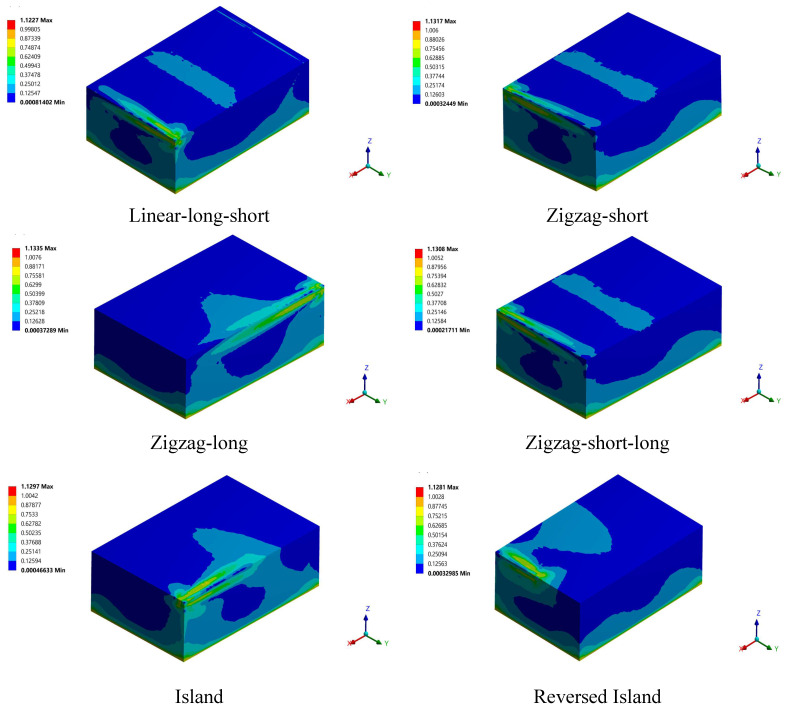
Normalized von Mises stress for the studied build ordinations just after solidification.

The von Mises equivalent stress normalization provides an accurate indication of the state of stress through the printed part. On the other hand, when the normalized von-Misses equivalent stresses are greater than or equal to one, the stress state is considered to be unsafe and there is a high possibility of crack formation. The stress state, on the other hand, is safe when the normalized von Misses equivalent stresses are less than one. Figure 13 shows the normalized von Mises stress (σ_e_/σ_y_) developed in the printed part at different build orientations. It can be observed from the stress contours that all the cases gave a normalized stress more than one. This means that there is a high possibility of crack formation for all the studied build orientations.

**Figure 14:**
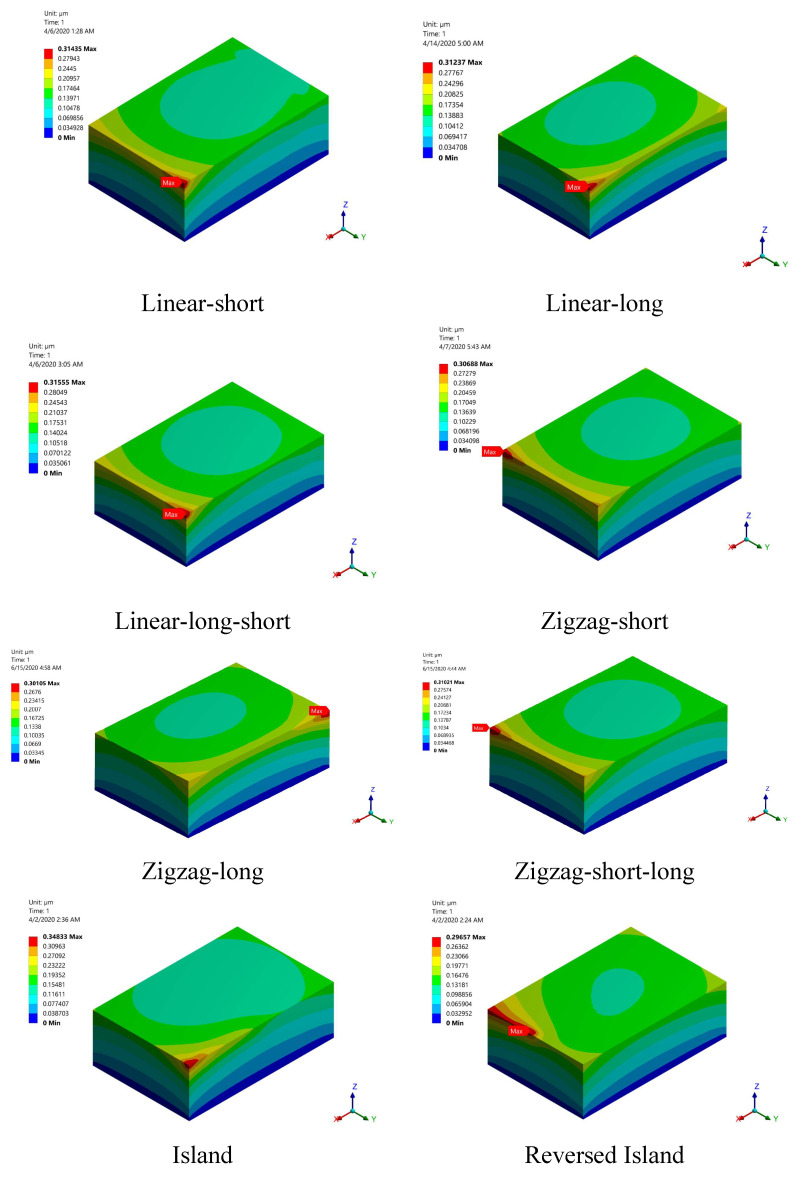
Distortion contours for the studied build ordinations just after solidification.

Figure 14 shows the distortion contours for the studied build orientations. The obtained distortion values are very small, in the order of 0.3 microns, and this is because of the small model dimensions (we could not consider large model dimensions as they require high computational capabilities, and one build orientation took almost one month to finish). It can be observed that the distortion values at the bottom of the part are very small, reaching zero, and increase gradually with the part’s height. This is because the bottom of the part (bottom layers) is attached to the base plate and has a temperature very close to room temperature. The upper layers are free to deform and have a higher temperature than the lower layers. The distortion value for all build orientations is very close, with the island scanning build orientation yielding the highest value.

**Figure 15:**
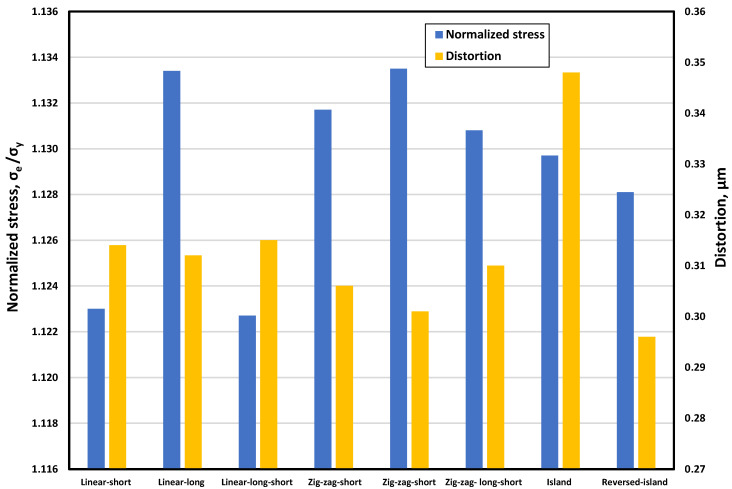
Residual stress and distortion for different build orientations.

Figure 15 depicts a bar chart for the normalized von Mises stress and distortion for the investigated build orientations. For the linear build orientations, the long-linear orientation had the highest residual stress, i.e., 13.3 percent above the yield limit, while the linear-short and linear-long-short orientation had the lowest residual stress, i.e., 12.2 percent above the yield limit. All of the linear build orientations produced nearly identical distortion results.

The zigzag build orientations resulted in a high residual stress of 13% above the yield limit, as well as a minor distortion lower than the linear build orientations. The island build orientations generated residual stress that was more than 12% higher than the yield. Additionally, the heat accumulation generated by the short scanning paths in a small area caused the island build orientation to have a high distortion value compared to the other build orientations. This trend did not appear in the reverse-island orientation, mainly due to the repeated layer scanning orientation changes.

Based on residual stress and distortion, it can be concluded that the linear-short and linear-short-long build orientations are the most effective.

### 3.5. Effect of Preheating

**Figure 16:**
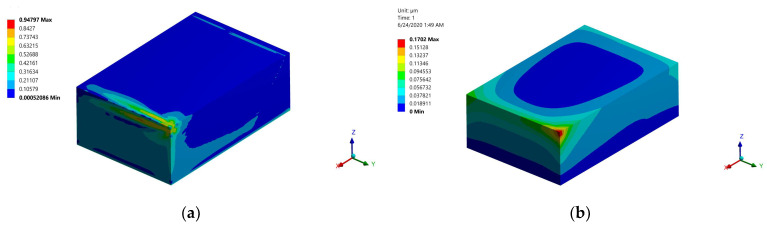
Normalized von Mises stress and distortion obtained using a preheating temperature of 800 K: (**a**) normalized von Mises stress; (**b**) distortion.

Preheating of 800 K was applied to the model for the linear-long-short build orientation. It can be seen from Figure 16a that the normalized von Mises stress decreased by 23% (from 1.22 to 0.947), and the developed distortion decreased by 54%. The reduction in developed stress and distortion is mainly due to the decrease in the temperature difference that the part has undergone. Therefore, it can be concluded that the PBSLP of alumina cannot be successful, i.e., free of cracks and defects, without preheating, and the available commercial printers cannot be used effectively for ceramic materials. Special printers equipped with a preheating system for ceramic materials are needed.

## 4. Conclusions

A multi-layer PBSLP model coupled with an FEA model was developed to simulate the real process of PBSLP and to investigate the appropriate selection of the process parameters, such as laser power, scanning speed, hatching distance, scanning strategies, and build orientations. Additionally, the model was used to investigate the effectiveness of using a CO_2_ laser for the PBSLP of alumina. The following points were concluded:

1. The model can be used to select the appropriate values of laser power, scanning speed, and hatching distance.
2. The CO_2_ laser can be effectively used for the PBSLP of alumina with a low power value and high scanning speed leading to a high manufacturing rate and energy conservation.
3. The model can obtain the printed part’s temperature history, temperature distribution, residual stress, and distortion.
4. The developed residual stress for the printed part exceeds the yield limit for all the studied build orientations. When preheating by 800 K is applied, the developed residual stress is reduced by 23% and the distortion by 54%. The linear-short and linear-short-long build orientations both gave low distortion and residual stress compared to other build orientations.
5. Commercially available printers cannot be used effectively for the PBSLP of ceramic materials, and a special printer equipped with a preheating system for the powder and temperature controller is needed.

For future work, the PBSLP of alumina using a preheating system should be considered.

## Figures and Tables

**Table 1:** Alumina material properties as a function of temperature and other constants.

Item	Symbol	Expression/Value	Unit	Ref.
Density	ρ	3920	kg/m^3^	
Specific heat ^1^	$C_{p}$	3 × 10^−13^ T^5^ − 3 × 10^−9^ T^4^ + 5 × 10^−6^ T^3^ − 0.0073 T^2^ + 5.0097 T − 190.71, (T ≤ 2450) 1360, (T > 2450)	J/kg-K	[42]
Thermal conductivity ^1^	K	−3 × 10^−15^ T^5^ − 3 × 10^−11^ T^4^ − 10^−7^ T^3^ + 0.0002 T^2^ − 0.203 T + 79.673, (T ≤ 2450) 5.5, (T > 2450)	W/kg-K	
Melting point,		2327	K	
Latent heat of melting,		1,137,900	J/kg	
Emissivity	ε	0.7		
Stefan Boltzmann constant,	σ	5.6704 × 10^−8^	W/m^2^ K^4^	
Thermal convection coefficient,	$h_{cov}$	200	W/m^2^ K^4^	
Absorptivity/CO_2_ laser	A	0.96		[39]
Absorptivity/Fiber laser	A	0.03		

^1^ The temperature is expressed in Kelvin.

**Table 2:** Numerical model geometry dimensions.

Dimension	Base Plate (mm)	Printed Part (mm)
Length	2	1.5
Width	1.5	1
Thickness	0.5	0.5 ^1^

^1^ The printed part contains 10 layers; each layer has a thickness of 0.05 mm.

**Table 3:** Mesh density analysis.

Mesh	Mesh Edge Size (µm)	Elapsed Time to Solve One Time Step (s)	Temperature (K)
A	10	3.6	3365.95
B	5	4.6	3388.48
C	2.5	5.7	3395.23
D	2	5.8	3396.35

**Table 4:** CO_2_ laser process parameters used in the study.

Item	Value
Laser power range, W	15–50
Scanning speed range, mm/s	500–1200
Layer thickness, µm	50

**Table 5:** Temperature distribution and melting contour obtained from the numerical model (Nd-YAG).

Power, W	Temperature Distribution	Top Melting Contour	Vertical Melting Contour
95	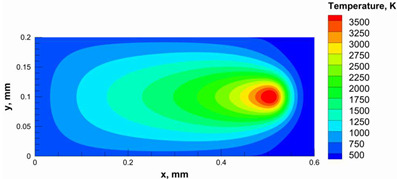	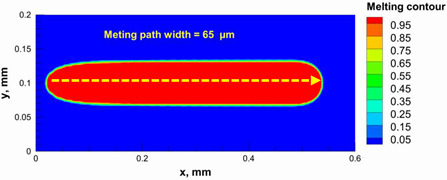	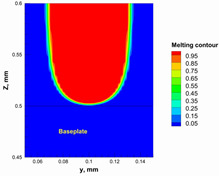
100	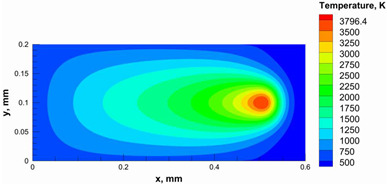	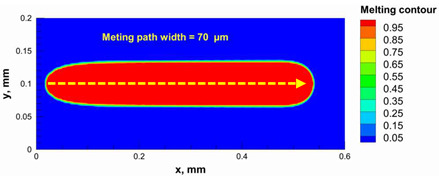	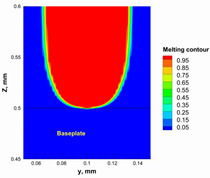
105	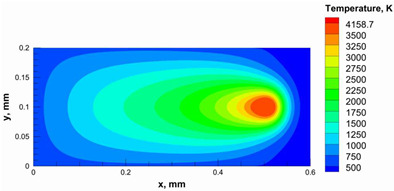	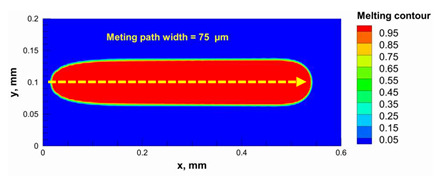	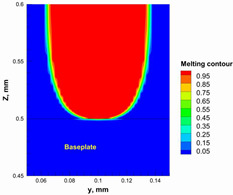

**Table 6:** Temperature distribution and melting contour obtained from the numerical model.

Power, W	Scanning Speed, mm/s	Temperature Distribution	Top Melting Contour	Vertical Melting Contour
30	600	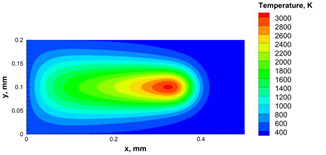	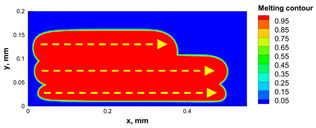	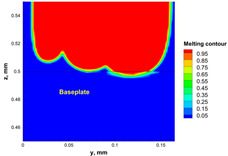
40	900	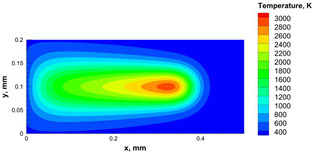	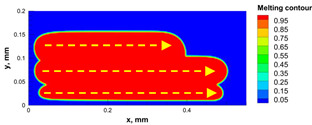	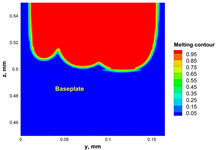
50	1200